# Breeding next generation tree fruits: technical and legal challenges

**DOI:** 10.1038/hortres.2017.67

**Published:** 2017-12-06

**Authors:** Lorenza Dalla Costa, Mickael Malnoy, Ivana Gribaudo

**Affiliations:** 1 Research and Innovation Centre, Fondazione Edmund Mach, via E Mach 1, San Michele a/Adige 38010, Italy; 2 IPSP-CNR, Institute for Sustainable Plant Protection, National Research Council, Strada delle Cacce 73, Torino I-10135, Italy

## Abstract

The new plant breeding technologies (NPBTs) have recently emerged as powerful tools in the context of ‘green’ biotechnologies. They have wide potential compared to classical genetic engineering and they are attracting the interest of politicians, stakeholders and citizens due to the revolutionary impact they may have on agriculture. Cisgenesis and genome editing potentially allow to obtain pathogen-resistant plants or plants with enhanced qualitative traits by introducing or disrupting specific genes in shorter times compared to traditional breeding programs and by means of minimal modifications in the plant genome. Grapevine, the most important fruit crop in the world from an economical point of view, is a peculiar case for NPBTs because of the load of cultural aspects, varietal traditions and consumer demands, which hinder the use of classical breeding techniques and, furthermore, the application of genetic engineering to wine grape cultivars. Here we explore the technical challenges which may hamper the application of cisgenesis and genome editing to this perennial plant, in particular focusing on the bottlenecks of the *Agrobacterium*-mediated gene transfer. In addition, strategies to eliminate undesired sequences from the genome and to choose proper target sites are discussed in light of peculiar features of this species. Furthermore is reported an update of the international legislative frameworks regulating NPBT products which shows conflicting positions and, in the case of the European Union, a prolonged lack of regulation.

## Introduction

In recent years a new generation of techniques, referred to as ‘new plant breeding techniques’ (NPBTs), emerged as powerful tool in the scenario of green biotechnologies all over the world, opening up a new breedomics era. From many points of view, their potential is by far wider if compared to that of traditional breeding and of transgenesis (i.e., ‘classical’ genetic engineering). These techniques are attracting the interest of politicians, stakeholders and citizens due to the revolutionary impact they may have on the agriculture of the future. The term NPBTs comprises several techniques, the best known being ‘cisgenesis’ and ‘genome editing through site-directed nucleases’.

The term ‘cisgenic plant’ was conceived in 2006^1^ and refers to a crop plant that has been genetically modified with one or more genes containing introns and regulatory sequences (promoter and terminator) in a sense orientation, isolated from the species itself or from closely related species capable of sexual hybridization. Furthermore, foreign sequences such as selection genes and vector-backbone sequences should be absent. Intragenesis differs from cisgenesis because it allows use of new gene combinations created by *in vitro* rearrangements of functional genetic elements.^2^ Although both transgenesis and cisgenesis use same molecular processes and techniques to transfer gene(s) into a plant, a cisgenic plant will retain only species-specific genes that could also have been transferred by traditional breeding.^3^ On the other hand, the introgression of desired genes from wild relatives (donor plant) into commercial varieties (recipient plant) through conventional breeding usually involves interspecific hybridization, followed by several generations of backcrosses with the recipient plant and simultaneous selection for the trait of interest. This can be achieved in a short time in annual crops, while in case of complex heterozygous, vegetatively propagated woody fruit crops with a long juvenile phase such as apple and grapevine, it requires a lapse of time that can last several decades and results in a genotype which can be quite different from that of the recipient plant. With the cisgenic approach only the genes of interest are permanently transferred in the recipient plant within a relatively short period of time.^4^

The genome editing approach was firstly used to knock-out undesired genes through the induction of DNA breaks at target sites by means of ‘guided’ endonucleases followed by the non-homologous end joining (NHEJ) repair process. This mechanism is responsible for the insertions or deletions of nucleotides at the target sites which may cause genetic mutations resulting in the silencing of the undesired gene. Among genome editing technologies, Zinc Finger Nuclease (ZFN), Transcriptional Activator-Like Effector Nuclease (TALEN) and Clustered Regularly Interspaced Short Palindromic Repeats (CRISPR), in order of appearance, are the most employed. All of them were optimized and refined at a very fast pace and rapidly spread all over the world as shown by the huge amount of published papers describing their application in different species, from model plants to herbaceous crops and fruit trees. Over the last years CRISPR/Cas9 system emerged as the most important tool for genome editing due to its simple structure and its applicability to a wide range of species.^5^ Compared to artificial mutagenesis, a common practice of conventional breeding which produces random mutations in the plant genome through application of chemical or physical agents, gene editing is a targeted mutagenesis approach able to recognize and modify a specific DNA sequence. The gene editing components may be delivered in plant cells by DNA vectors following processes similar to those used in the transgenic approach or, as an alternative, systems based on ribonucleotide-protein complexes used to produce non-GM edited crops.^6^

A synthetic analysis of the strengths and weakness of traditional and new breeding techniques is reported in Table 1.

## New achievements in plant breeding by NPBTs: will they be welcome? how should they be regulated?

A long road has been travelled since the first attempts of breeding made by ancient farmers who selected best phenotypes to propagate without knowing the genotype. After a long period of traditional breeding achieved mainly by controlled crosses and through selection of spontaneous or induced mutants, the advances of molecular genetics have strongly impacted on the potentialities and achievements of modern breeding. The transgenic technology allows to transfer a desired cloned gene, and genetically engineered cultivars of many crops have been obtained, commercialized and cultivated all over the world in the last decades (http://www.isaaa.org/). Nevertheless, this technique not only faces technical challenges as many economically important species or élite cultivars proved recalcitrant to gene transfer and/or regeneration, but also it has risen a great deal of ethical criticisms. The public acceptance of transgenic crops appears to be much higher in USA than in Europe, and the rules regulating their cultivation and commercialization are still quite different. USA has a product-oriented approach, which allows to approve a transgenic cultivar if it has a substantial equivalence with a conventional crop, while in the European Union the current legislation is process- or technique-oriented with emphasis on the precautionary principle. The topic has been widely discussed at political level and on the media, involving fear of unpredictable risks. Differences between the two approaches have been described and analyzed by several authors.^7,8^ Countries as Canada, Argentina, Japan and India have genetically modified organism (GMO) regulations similar to that of USA, while Australia, New Zealand and China regulate GM crops with various levels of restriction. As of September 2017, the Cartagena Protocol on Biosafety (an international agreement to ensure the safe handling, transport and use of living modified organism generated by modern biotechnology; https://bch.cbd.int/protocol/) has not yet been ratified by countries such as Argentina, Australia, Canada and USA. In Europe, public perception of a GMO largely depends on its purpose: transgenic animals or plants to be used for feed or food have failed in gaining public acceptance, while few objections have been raised on the use of GMOs for medical or pharmaceutical aims. In addition, traceability is a current topic in Europe and this involves also products deriving from GMOs. The current EU legal framework (https://ec.europa.eu/food/plant/gmo/legislation_en) requires clear labelling of GMOs placed on the market; the labelling requirements do not apply to GM food/feed products in a proportion no higher than 0.9% of the food/feed ingredients considered individually and if this presence is adventitious or technically unavoidable. At this moment meat from animals fed with transgenic fodder does not need a specific label.

Also, in USA more attention is now paid to ethical issues and to traceability, as showed by the Vermont case. The Vermont state promulgated a law (Act 120, effective 1 July 2016) which requires the labeling of food produced entirely or in part with genetic engineering (http://www.natlawreview.com/article/reminder-vermont-gmo-labeling-law-vermont-act-120-goes-effect-july-1). Afterwards, on 29 July 2016, President Obama signed into law legislation that creates a nationwide mandatory labeling regime for GMOs in foods. The law directs the Agriculture Department (USDA) to establish, within two years, a national process to identify GMO food products or ingredients that should be disclosed. The legislation will require food packages to display an electronic code, text label, or some symbols signifying whether or not they contain GMOs (http://www.natlawreview.com/article/president-obama-signs-gmo-labeling-bill-law). Although controversial—this law was accused to act in the interests of GMO producers—it would set a national standard for labeling products with GMOs. In Canada an initiative similar to the Vermont one was launched although unsuccessfully. In China the government supports biotech but public opinion is very sensitive to food safety issues and somehow succeeded in slowing down transgenic food approval and diffusion (http://www.newyorker.com/tech/elements/can-the-chinese-government-get-its-people-to-like-g-m-o-s).

In this framework, the new plant breeding technologies have raised much attention, as these approaches not only have highly interesting potentialities in breeding but also could overcome many ethical restraints, being techniques that mimic spontaneous events. As for classical GMOs, also for the NPBT products the regulatory paradigms of nations focus on the process used, like in Europe at present, or on the nature of the novel phenotype developed. In USA the Department of Agriculture Animal and Plant Health Inspection Service (USDA-APHIS) stated that plants derived from cisgenesis/intragenesis or modified with ZFNs and TALENs are not considered regulated articles as they do not contain foreign DNA from plant pest.^9^ Recently, in April 2016, the common white button mushroom (*Agaricus bisporus*) resistant to browning was the first CRISPR-edited organism to receive a green light from the US government.^10^ In Australia cisgenic plants are excluded from GMO legislation while in Argentina, where the world’s first regulation for NPBT was issued in 2015, products without transgenes are to be evaluated on a case by case basis.^11^ In the European Union there is a great deal of uncertainty and the debate on the legal interpretation of genome editing techniques is extremely lively. European regulatory experts and scientists carefully explored all the features and elements of novelty carried by the new plant breeding technologies.^12,13^ In 2012, the European Food Safety Authority (EFSA) issued two scientific opinions on the new breeding techniques: on the safety assessment of plants developed by cisgenesis and intragenesis^14^ and another on the safety assessment of nuclease-based genome editing.^15^ EFSA concluded that the existing guidelines for risk assessment applicable to GM plants were also appropriate for cisgenic and intragenic plants, and for the ZFN-3 technique. EFSA also considered the hazards associated with cisgenic plants to be similar to those linked to conventionally bred plants, but that novel hazards could be associated with intragenic and transgenic plants.

**Figure 1:**
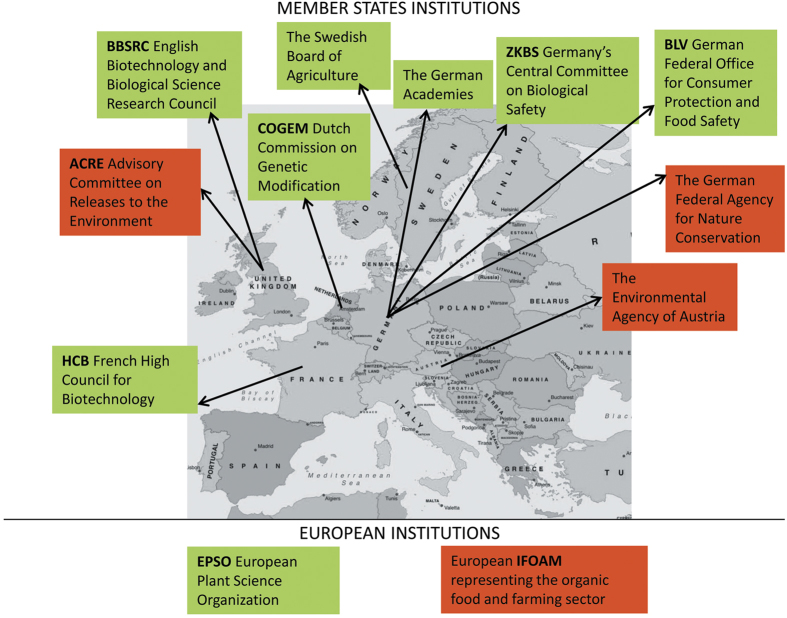
Main national and European institutions claiming that the products of NPBT (cisgenesis and/or genome editing techniques) should fall (red boxes) or not (green boxes) under GMO legislation.

On an institutional front, opposing positions have been taken by different European national bodies (Figure 1). The English Biotechnology and Biological Science Research Council (BBSRC), the German Academies, the European Plant Science Organization (EPSO) and the French High Council for Biotechnology (HCB) consider that the safety of new crop varieties should be assessed according to their characteristics rather than the method by which they are produced.^16^ The Dutch Commission on Genetic Modification (COGEM) pointed out that cisgenic products should be exempt from GMO legislation; Germany’s Central Committee on Biological Safety (ZKBS) and the German Federal Office for Consumer Protection and Food Safety (BLV) considered organisms modified by genome editing technologies as not being GM and the Swedish Board of Agriculture concluded that CRISPR/Cas9 should not be subjected to European GMO legislation.^16^ On the other side, national environmental agencies (the UK Advisory Committee on Releases to Environment—ACRE, the German Federal Agency for Nature Conservation and the Environmental Agency of Austria) as well as the European IFOAM representing the organic food and farming sector, were more prone to consider these products as GMOs on the basis of a precautionary principle.^16^

In 2013, the European Academies Science Advisory Council (EASAC) came to the conclusions that ‘the trait and product, not the technology, should be regulated, and the regulatory framework should be evidence-based’.^8^ This report was endorsed by several academic organizations, most prominently by Anne Glover, former Chief Scientific Adviser to the President of the European Commission (EC) who stated: ‘We shouldn’t forget that there are also other promising novel plant breeding technologies, post-GM, and we shouldn’t make the mistake of regulating them to death as we have done with GM’.^8^ Two years later the same EASAC argues that the products of NPBTs should not fall under GMO legislation when they do not contain foreign DNA and demands EU regulators to resolve current legislative uncertainties by modernizing the present regulatory framework.^8^

To date (September 2017), a clarifying legal opinion of the EC is still pending and probably will follow the sentence of the European Court of Justice (ECJ) which was asked on October 2016 by French national authorities to rule on whether the new biotechniques for targeted mutagenesis can be exempted from GMO legislation such as the procedures based on chemical and physical mutagenesis. The judgement is expected within the first semester of 2018 and it will be mandatory for all the member states.^17^

On 28 April 2017 the High Level Group of the European Commission's Scientific Advice Mechanism (SAM) published an independent explanatory note on ‘New Techniques in Agricultural Biotechnology’ ^13^ following the request of Vytenis Andriukaitis, European Commissioner for Health and Food Safety. According to the available scientific reviews, expert opinions and reports, the document describes and compares the new techniques with conventional breeding techniques and with established techniques of genetic modification. The Commissioner said that this document will be an important scientific basis to stimulate an informed public debate among all stakeholders addressing the challenges and opportunities related to innovation in the agro-food sector (https://ec.europa.eu/research/index.cfm?pg=newsalert&year=2017&na=na-280417).

In Italy, in February 2016 the Agriculture Minister Maurizio Martina made a distinction between innovative biotechnologies and GMOs and advocated innovation involving cisgenesis and genome editing.^18^ The Minister Martina stated his support for these two technologies by allocating 21 million of euros in Italy’s budget for a three-year sustainable agriculture research plan to be implemented by the Italian Council for Agricultural Research and Economics (CREA). The research will focus on genome editing and cisgenesis for grapevine, olive, apple, citrus fruit, apricot, peach, cherry, pineapple, tomato, wheat and poplar.^18^

## NPBT’s challenges in grapevine and other fruit trees

The application of cisgenesis and genome editing to perennial fruit trees faces many challenges compared to species propagated by seed as proven by limited scientific literature available for woody fruit crops (Table 2). A crucial issue to consider concerns the elimination of undesired exogenous sequences from the plant genome. The absence of the marker gene is mandatory in the case of cisgenesis, likewise the T-DNA used to deliver gene-editing components should be removed in view of leaving minimal genetic modifications. While for annual plants these sequences can be eliminated by self-fertilization and segregation in the first generation of offspring, in case of vegetatively propagated woody fruit crops this strategy would encounter the same problems that hamper the cross-based traditional breeding (long generation time, offsprings which can be genetically and phenotypically quite different from the parental plants). This limit has led to the development of alternative approaches to remove undesired sequences.^19^

In this review, we discuss different aspects of the application of cisgenesis and genome editing to fruit trees referring in particular to grapevine, not only for the enormous economic value this crop has worldwide, but also for its peculiar features which make it an interesting case-study for NPBTs. Among grapevine genotypes a distinction should be made: while breeding of grapevine for table grape production is rather similar to that of fruit crops such as apple, pear and others, the use of classical techniques as well as the application of genetic engineering to wine grapes has been hindered by a load of cultural aspects, varietal traditions, consumer demands and regulatory framework (particularly in the Old World). In fact, on one side the wine industry relies predominantly on a few selected and sought-after cultivars whose features would be altered by crossing in breeding programs; on the other side, the concept of genetic manipulation performed in laboratory in order to introduce desired traits in centuries-old elite cultivars has so far been hardly accepted by oenophiles and consumers fond of the view of wine as a natural product. The genetic improvement of this valuable fruit crop may gain a great benefit from these new technologies which resemble traditional breeding techniques but require shorter times and do not alter the genetic heritage of the cultivar of interest. It has to be taken in account that many efforts have already been made to explore the grapevine genome, looking for interesting genes to transfer with both traditional and biotechnological tools. An interesting case of immediate application of cisgenesis in *Vitis vinifera* may be the transfer of pathogen resistance genes. Single locus genes which confer resistance to the major fungal and oomycete pathogens in cultivated grapevine (powdery and downy mildew) were well characterized and may be transferred by a donor to a recipient grapevine. Among them, there are MrRUN1 and MrRPV1 which were isolated from *Muscadinia rotundifolia,* a wild north American grapevine species.^20^ An interesting future application of genome editing in grapevine may be the silencing of susceptibility MLO genes whose knock-down has been demonstrated to confer resistance to powdery mildew.^21^

**Figure 2:**
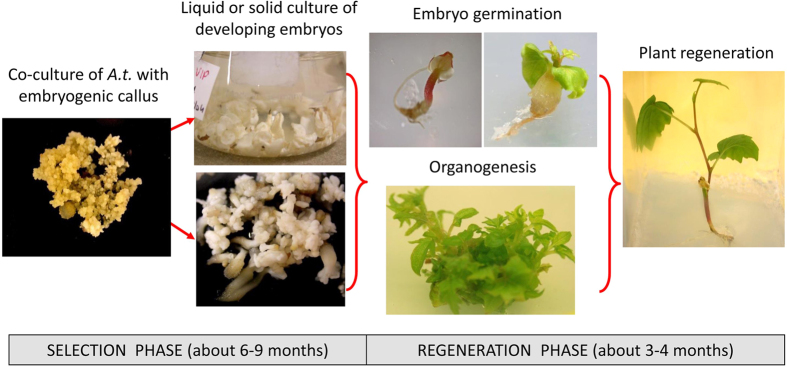
Workflow of a gene transfer process in grapevine via *Agrobacterium tumefaciens*. The selection phase is carried out in the presence of selection agents (i.e., antibiotics or herbicides).

The *Agrobacterium*-mediated gene transfer (Figure 2) has emerged as the most widely used method in plant genetic engineering, although manifold systems for these new technologies have been developed based on different constructs, delivery and expression mechanisms.^6^ However, the setting-up of efficient transformation procedures in woody fruit trees requires to optimize several technical aspects concerning tissue culture. One of the main limiting factors, common to most of the perennial fruit crops, is the limited regeneration capability of the explant used (i.e., somatic embryos for grapevine, leaves for apple and pear) in co-culture with *Agrobacterium*.^22,23^ Looking specifically to grapevine, the main critical points regard the ability to produce embryogenic callus, the response of the callus to *Agrobacterium tumefaciens* infection, the regeneration potential of somatic embryos, the chimerical integration of exogenous DNA and somaclonal variation as an outcome of tissue culture. Other issues such as strategies to eliminate undesired sequences from the genome and to choose proper target sites are essential, as well as proper analytic tools to characterize the results.

### Technical aspects of *Agrobacterium*-mediated gene transfer. Grapevine as a case study

#### Recalcitrance of different genotypes at producing embryogenic callus

Embryogenic callus is the most used explant for gene transfer experiments in grapevine.^24,25^ This is confirmed by the use of embryogenic culture of ‘Chardonnay’ in the first study describing genome editing and targeted mutation in grapevine.^26^ In a recent paper^27^ the CRISPR/Cas9 system was successfully applied to produce point mutations in grapevine protoplasts DNA. However, the regeneration of a plant from a grapevine protoplast is very difficult to obtain and nearly unfeasible for many cultivars. The isolation of protoplasts from embryogenic grapevine tissue and the regeneration of these protoplasts into plants were successful only with two *Vitis vinifera* cultivars, ‘Seyval blanc’^28^ and ‘Koshusanjaku’.^29^ Some authors ascribed the regeneration recalcitrance of grapevine protoplasts to the lack of morphogenic response *in vitro*.^30^

Some varieties displayed embryogenic competence while others proved to be recalcitrant, and wide variations among responsive varieties were observed.^31,32^ Factors influencing somatic embryogenesis include explant type and developmental stage, macro- and micro-element composition of the culture medium and growth regulator concentration.^32,33^ According to several studies, a greater number of *Vitis vinifera* varieties produced embryogenic cultures from stamens and pistils compared with leaves.^34^ Dhekney *et al.*,^32^ among the 19 cultivars and 3 rootstocks evaluated, observed the highest embryogenic response from ‘Merlot’ stamens and pistils (11.6±0.2% and 13.8±0.3%) followed by ‘Thompson Seedless’ (10.5±0.2% and 8.3±0.3%) and ‘White Riesling’ (3.7±0.2% and 5.9±0.3%). Gribaudo *et al*.^35^ evaluated the embryogenic competence of 38 grapevine cultivars and rootstocks over many years and identified genotypes with high regenerative competence like ‘Chardonnay’, ‘Müller Thurgau’, ‘Sangiovese’ (which showed an efficiency of 10% with anther cultures and/or 20% with ovary cultures) as well as recalcitrant ones such ‘Cabernet Sauvignon’ (efficiency below 1 and 2% in anther and ovary culture respectively). However, as the authors stated, the results for a same cultivar can vary in different years, suggesting that the complete control of all the factors influencing the embryogenic induction is hard to reach.

Various genes playing a role in regulating the somatic embryogenesis process in grapevine as well as in other species have been identified such as SERK, L1L^36,37^ and WOX.^38,39^ It can be hypothesized that they could allow improvement of the transformation methodology if transferred together with the gene of interest and expressed in calli or somatic tissues of recalcitrant grapevine cultivars, thus inducing the differentiation of somatic embryos.

#### Response of the callus to *Agrobacterium tumefaciens (A.t.)* infection

Since *Agrobacterium vitis* is a specific pathogen of *Vitis vinifera*, grapevine embryogenic cultures inoculated and co-cultivated with *Agrobacterium* ssp. can show an ipersensitive reaction and tissue browning^40^ albeit the severity of the reaction is strictly cultivar specific. The ‘Pinot’ family for example proved intractable to *A.t.* mediated transformation while genotypes like ‘Chardonnay’,^41^ ‘Brachetto’,^42^ ‘Portan’^43^ and ‘Thompson seedless’^44^ showed high efficiency. Moreover, the necrosis phenomenon is not only cultivar specific, but also developmentally regulated since has been demonstrated that embryogenic cells in a more advanced stage of embryo development exhibited more severe tissue necrosis than cells of pro-embryogenic masses.^45^ The necrosis typically occurs 48 h onwards after co-cultivation and can in some cases be so severe that the target material never becomes proliferative again.^46^ Zhao *et al.*^47^ demonstrated that this response is due to the downregulation of enzymes involved in reactive oxygen species (ROS) removal, up-regulation of ROS producers and significantly changed levels of plant-pathogenic response proteins. Specific protocols have been established for different species and varieties, tissue-culture media have been carefully optimized, and additional antioxidants, active charcoal and washing steps have been employed to decrease the observed tissue necrosis.^47,48^

#### Regeneration potential of somatic embryos

The regeneration potential of a somatic embryo, i.e., the capability of the somatic embryo to convert into a plantlet, is a peculiar feature associated to a specific variety. However, an important aspect affecting the regeneration potential of somatic embryos is the age of the callus. A fresh embryogenic callus (1 or 2 years of age) shows a high morphogenic competence and assures embryo development and conversion into plantlet at a high rate. However, a possible drawback of using a young callus is the high likelihood of finding ‘escapes’ due to the regeneration of plantlets which have not integrated the exogenous DNA but have survived to the selection regime, probably thanks to a lower sensitivity towards the selection agent associated with the strong morphogenic potential. There are other important factors influencing the regeneration potential of somatic embryos like callus sensitivity to the antibiotics used as selection agents or to *Agrobacterium* killing agent. Also *Agrobacterium* strains and density used to infect the embryogenic callus may impinge on the regeneration potential.^49^

#### Chimerism

One of common technical constrains of gene transfer into vegetatively propagated plants is the chimerical integration of the exogenous DNA in the plant tissues. The regeneration of chimeric shoots with heterogeneous tissues made up of mixtures of transgenic and non-transgenic cells has frequently been reported in herbaceous and perennial crops.^50,51^ However, while chimerism can be eliminated in the progenies of a sexually propagated plant, the complete loss of chimerism is difficult to obtain for vegetatively propagated plants like grapevine and only possible by using *in vitro* culture procedures like further embryogenesis or organogenesis. The presence of chimerism may be an additional obstacle for obtaining genome edited plants since the partial efficiency of the CRISPR/Cas9 system may be further reduced by a potential chimerical distribution in the plant tissues.^52^ Therefore, it is not surprising that in the first study on genome edited grapevine the 100% of cell mass are mosaics and the harvested plants might be heterozygous or chimeras.^26^ This study described the use of the CRISPR/Cas9 system for silencing the L-idrate deidrogenase (*IdnDH*) gene in grapevine. The authors infected ‘Chardonnay’ embryogenic calli with *Agrobacterium tumefaciens* carrying a vector with the Cas9 gene sequence, the guide RNA and the selectable resistance marker gene *hpt* which confer resistance to hygromicin. Plants with insertion or deletion in *IdnDH* were regenerated with an efficiency of 5% or 0% in two different experiments (where efficiency is calculated as n. of lines with targeted mutations / n. of cellular masses resistant to hygromicin). The ‘edited’ plants however, were all heterozygous or chimeric.

#### Somaclonal variation

Tissue culture is an efficient method of clonal propagation, however the resulting regenerants can exhibit somaclonal variations.^53^ This variation involves changes in both nuclear and cytoplasmic genomes, and their character can be of genetic or epigenetic nature.^54^ The triggers of mutations in tissue culture had been attributed to numerous stress factors, including wounding, exposure to sterilizing agents, imbalances of media components such as high concentration of plant growth regulators (auxin and cytokinins), sugar from the nutrient medium as a replacement of photosynthesis in the leaves, lighting conditions, the disturbed relationship between high humidity and transpiration.^55,56^ The rate of somaclonal variation can be particularly high when somatic embryos are induced in callus tissue, in a long-term cultures,^54^ or via secondary embryogenesis.^57^ On the contrary, direct development of somatic embryos from cultured explants and/or the use of young explant tissue in combination with short term culture usually limit *in vitro* induced variation.^58^ While somaclones regenerated from callus cultures possibly may be a source of variation useful for plant breeding, for applications like micropropagation and genetic transformation it is essential to eliminate or decrease somaclonal variation.

In grapevine, somaclonal variation is frequently observed among plants regenerated through somatic embryogenesis.^59^ A wide range of traits showing somaclonal variation has been described such as chlorophyll deficiencies, morphogenetic development, leaf shape, flower type ^60,61^ but no grapevine cultivar derived from somaclonal variation has been so far released. By using SSRs, AFLPs (amplified fragment length polymorphism) and RAPD (random amplified polymorphism DNA) hundreds of somaclones obtained from different grapevine cultivars were analysed to determine the level of genetic variation.^62,63^ SSRs were useful to verify the conservation of the microsatellite profile of the somaclones as to their corresponding mother clone genotype. Among studies performed using SSRs, only one reports the variation of one SSR in 6 plants out of 233 regenerated from somatic embryo.^64^ On the contrary, more variation was observed with AFLP markers in the somaclones.^63^

Differences between clones can also result from epigenetic modifications like DNA-methylation ^65^ which can generate novel and heritable phenotypic variations.^66^ Epigenetic variations are suggested to be more frequent than genetic changes under *in vitro* conditions.^67^ Apart from auxins, other *in vitro* employed substances can also influence the level of DNA methylation, as was indicated for antibiotic in callus culture of *Arabidopsis*.^68^ The methylation sensitive amplified polymorphism (MSAP) method is used by many authors to identify genomic regions with altered 5-methylcytosine distribution at a genome-wide scale.^69^ The MSAP analysis carried out in somaclones of two *Vitis vinifera* cultivars (‘Chardonnay’ 96 and ‘Syrah’ 174) regenerated from somatic embryos revealed methylation status variation compared to the mother clone.^63^ Ocana and colleagues ^70^ evaluated the epigenetic variation in a set of 40 ‘Pinot noir’ clones using the MSAP technique and identified stable epigenetic markers suitable for clone selection.

### Removal of exogenous sequences

Cisgenic plants should be free from additional sequences, such as selectable marker genes. To date, it has been demonstrated that although it is achievable to obtain modified plants without using selection, the majority of the screened plants would be un-transformed making the screening process excessively demanding and laborious.^19^ At the same way, in order to increase the acceptability of gene-edited plants, no exogenous sequences (e.g., T-DNA containing the cassette with Cas9 endonuclease, the sgRNA and the selection marker) should remain in the plant genome after the desired mutation has been carried out. In this respect, the removal of the T-DNA is important not only for producing transgene-free edited crops but also because the persistent Cas9 nuclease activity may increase the likelihood of off-target effects (cleavage and mutation at unintended genomic sites similar but not identical in sequence to the desired site).^71^ The feasible solution seems to be the use of site-specific recombination systems relying on the activity of a recombinase enzyme which recognizes two directly repeated sites and produces the excision of the DNA cassette in the middle.^19^ The most frequently used recombinase/recognition sites are the bacteriophage P1 Cre/loxP, the yeast Flp/FRT,^72,73^ and the R/Rs from *Zygosaccharomyces rouxii.*^74^ Since the excision timing needs to be controlled, recombinase gene expression should be regulated by inducible promoters, the majority of which are chemically activated, tissue specific or heat-shock activated.^75^ An interesting approach to minimize the risks of off-targets effects may also be the use of an inducible T-DNA excision associated with an inducible promoter to drive Cas9 expression. These excision systems are very precise and effective ^76^ but need to be *ad-hoc* developed and optimized for each species. However, while site-specific recombination systems were tested and used to remove marker resistance genes in fruit trees (Table 2), their effectiveness to excise longer regions like T-DNA has not yet been demonstrated. Regarding grapevine, during a proof-of-concept study, Dalla Costa *et al*.^76^ integrated a reporter gene in grapevine ‘Brachetto’ plants adopting a traditional selection with kanamycin and subsequently removing the *nptII* marker gene with a site-specific and inducible recombination mechanism. These authors showed that after a proper heat-shock induction no traces of *nptII* remained in the grapevine tissues and that the excision mechanism at the nucleotide level was highly accurate and precise. Conversely, the excision induced by the hormone 17-β-estradiol proved to have different efficiencies in the various tissues of ‘Brachetto’ transgenic plants, being very effective in the roots while only partially effective in the apical parts like leaves, nodes and internodes although different hormone supply strategies were performed.^77^ In view of producing a marker-free grapevine, also the co-transformation system, associated with a combination of positive or negative selection, has been successfully employed in 'Thompson Seedless' ^78^ but being this method highly dependent on an efficient regeneration, it might not be extended to many *Vitis* genotypes.

### Choice of the target site for genome editing

An important aspect to take in consideration for the choice of the target sequence is the genome heterozygosity of the species of interest and the degree of intra-specific genetic variation. In this regard, grapevine is an interesting case showing a highly heterozygous genome and a high genetic variation among cultivars and accessions. From the comparison of the coding region of single copy genes (for a total length of >3000 bp) in 157 cultivars of *Vitis vinifera,* a total of 96 polymorphic sites were recorded with an average frequency of 1 SNP/34.55 nucleotides.^79^ Several tools are publicly accessible on the web for designing sgRNAs that target unique locations in the plant genome^80,81^ and a recently developed database^82^ for facilitating the use of the CRISPR/Cas9 system is available for public use at the Grape-CRISPR website (http://biodb.sdau.edu.cn/gc/index.html). For selecting the best target sequences according to the criteria required by the different systems (ZFN, TALEN, CRISPR) the grapevine reference genomes^83,84^ can be used. However, after this step, a subsequent sequencing of those regions in the genotypes of interest is needed to avoid bumping into SNPs which can prevent an optimal recognition by the endonuclease and consequently the DNA cleavage.

### Analytical tools

The availability of suitable tools for the molecular characterization of GM products is essential, especially in the European Union which has highly restrictive rules concerning GMOs authorization. At the moment, a genetically modified organism can be put on the European market after it has been authorized by the European Commission on the basis of a detailed application procedure that is described in European Regulation EC 1829/2003. The technical dossier of an application must be compiled according to Commission Implementing Regulation EU No 503/2013 and to EFSA’s guidelines. It dedicates an important section to the molecular characterization of the GM products. The list of the required information includes: description of the methods used for the genetic modification, nature and source of vector used, source of donor nucleic acid(s) used for transformation, size and intended function of each constituent fragment of the region intended for insertion, general description of the trait(s) and characteristics, which have been introduced or modified, information on the sequences actually inserted/deleted, information on the expression of the insert(s), genetic stability of the insert and phenotypic stability of the genetically modified plant.

In the case of a cisgenic plant an important information on the sequences actually inserted is their copy number. According to the scientific literature on this topic^85^ and in view of minimally alter the plant genome, a single integration event is highly recommended. The determination of the copy number relies on the traditional Southern blot technique and the quantitative PCR on genomic DNA. Moreover, the complementation of these two assays can reveal the presence of chimeric tissues as described in Dalla Costa *et al.*^51^ Besides, very important is the determination of the removal rate of the undesired sequences such as the selectable marker gene and the components of the excision-cassette. A proper induction method has to be set up to find the optimal conditions for a complete excision of the exogenous sequences since a cisgenic plant must not contain them by definition. The available technique to assess the percentage of removal is the qPCR on genomic DNA.^76^ Another aspect which should be investigated is the integration point of the inserted sequence in the grapevine genome which can heavily influence its expression pattern (position effect). Higher plant genomes contain a substantial amount of intercalary heterochromatin and repetitive DNA, in addition to centromeres and telomeres, which can exert a repressive influence on a transgene inserted in their proximity.^86,87^ Analytical assays exist, such as chromosome walking or genome walking, based on digestion of genomic DNA, ligation of the DNA fragment ends with adaptor, PCR and sequencing. To this purpose, commercial kit are available on the market. Moreover, for a highly detailed and comprehensive knowledge of the cisgenic plant genome a whole genome sequencing (WGS) may be carried out. WGS could precisely identify the location of the inserted gene and check the presence of possible undesired truncated integration fragments which may be generated when using the biolistic technology (and less with the gene transfer via *Agrobacterium*).

Regarding the molecular characterization of genome edited plants, first of all, an estimation of the mutation rate is needed. Since the CRISPR-Cas9 system has been the most widely adopted technology in recent years in medical and plant research, hereafter we will refer to it when talking of the genome editing approach. As discussed above, T-DNA chimerical integration is a common outcome of the gene transfer process in grapevine. In addition the efficiency and timing of the genome editing system may be highly variable. For example, when a sequence is mutated after the division of the first embryogenic cell, the resulting cells of the somatic embryos have different genotypes and the regenerated plants exhibit different chimeric phenotypes.^52,88^ In order to determine the mutation rate and the kinds of mutation (big or small insertion/deletions—INDEL), a PCR can be performed for the amplification of a region containing the target sites. The amplification fragments can be separated on a high density agarose gel for a raw discrimination of large-sized mutations. However, for the detection of small INDEL mutations, the cloning of the PCR product in a vector, its insertion in *Escherichia coli* cells and the sequencing of an appropriate number of colonies is necessary. A fast and cheap alternative method to distinguish small INDEL mutations relies on the disruption of a restriction site positioned near to the Protospacer Adjacent Motif (PAM) site since the Cas9 cuts 3–4 bp upstream of the PAM sequence.^89^

Finally, the sequencing of specific regions characterized by a high level of similarity with the target site would allow to find possible off-target mutations. However, for an exhaustive and definitive off-targets check a WGS may be required.

When characterizing a transgenic plant, besides the analyses needed to ascertain the various molecular features resulting from the gene transfer, high-throughput phenotyping can be used for the evaluation of transgenic plants. Imaging methodologies are used to collect data for quantitative studies of complex traits related to the growth, yield and adaptation/resistance to biotic or abiotic stress. These techniques include visible imaging, imaging spectroscopy, thermal infrared imaging, fluorescence imaging, 3D imaging and tomographic imaging.^90–92^ In grapevine, automated phenotyping approaches have been up to now proposed to increase objectivity, automation and precision of data collected in vineyard.^93,94^ The adoption of such high-throughput techniques could help to characterize the phenotype of transgenic grapevines in controlled environment, when stringent biosecurity measures restrict the feasibility of open field trials.

## Conclusions

The scientific and technological progresses are undoubtedly key factors to obtain genetically improved grapevine derived from the new plant breeding technologies, which have remarkable potentialities. The availability of the sequenced genomes for several grapevine cultivars is giving further stimulus to the researches in this field. However, an updating of the legislative framework and an enhanced public acceptance based on a better understanding of the topic are pivotal for future turning of the scientific improvements into practical applications in breeding programs. Further and stronger efforts in all these fields are needed.

## Figures and Tables

**Table 1:** Comparison of the main features of traditional and new breeding techniques for woody fruit trees

	*Hybridization*	*Chemical or physical mutagenesis*	*Transgenesis*	*Cisgenesis*	*Genome editing (for inducing nucleotide modification)*
Method of gene transfer/modification	Controlled crossing	Exposure to chemical or physical agents	Gene transfer, mainly through *Agrobacterium tumefaciens* (T-DNA integration) and biolistic method	Gene transfer, mainly through *Agrobacterium tumefaciens* (T-DNA integration) and biolistic method	Gene modification, mainly through *Agrobacterium tumefaciens* (T-DNA integration) and ribonucleoproteins (RNPs)
Origin of the genes introduced or modified	Plant of the same species or of a crossable species	Plant itself (endogenous genes)	Any organism	Plant of the same species or of a crossable species	Plant itself (endogenous genes)
Targeted vs random approach	Choice of the parents is crucial, while the genetic mixing is random. The desired introgression can be identified through a selection process	The mechanism is untargeted. The desired mutation can be identified through a selection process	Gene of interest (GoI) is integrated in the plant genome but the integration point is untargeted	GoI is integrated in the plant genome but the integration point is untargeted	The nuclease cleavage is targeted while the T-DNA integration is untargeted
Use of selectable marker genes (SMG)	No	No	Yes	SMG can be used but must be removed	SMG must be removed
Involvement of genes other than GoI	Yes	Possible	Low risk	Low risk	Low risk
Possibility to distinguish from a natural occurrence	No	No	Yes	Yes	No (if the T-DNA is completely removed)
Time-consuming drawbacks	Several time-consuming backcrossings may be needed to achieve the desired goal	Mutation type and stability must be controlled on a several year-long period	Plants can be obtained in a relatively short time, but must undergo approval procedures	Plants may be obtained in a relatively short time provided the protocol has been set up; the approval procedures are to be defined in EU and other countries	Plants may be obtained in a relatively short time provided the protocol has been set up; the approval procedures are to be defined in EU and other countries

**Table 2:** Applications of cisgenesis and genome editing to woody fruit trees

*CISGENESIS*			
*Species*	*Gene of interest*	*Method for marker gene elimination*	*References*
Grapevine (*Vitis vinifera* L.)	A reporter gene was used to set-up the method	Site-specific recombination (Flp/*FRT*) induced by heat treatment	76
	Different grapevine promoter were proposed for an intragenic approach	—	95
	*	Site-specific recombination (Cre/*LoxP*) induced by 17-β-estradiol	77
Apple (*Malus* x *domestica* Borkh.)	*MdMyb10* which confers a red pigmentation	No use of marker gene	96
	*Vf* (*Rvi6)* from *Malus floribunda* 821 which confers apple scab resistance	Site-specific recombination (R/*Rs*) induced by dexamethasone followed by selection on 5-fluorocytosine	97 96
	*Vf* (*Rvi6)* from *Malus floribunda* 821 which confers apple scab resistance	Site-specific recombination (Flp/*FRT*) induced by heat treatment	98
	FB_MR5 from *Malus×robusta* 5 which confers fire blight resistance	Site-specific recombination (Flp/*FRT*) induced by heat treatment	99
	A reporter gene was used to set-up the method	Site-specific recombination (R/*Rs*) induced by dexamethasone followed by selection on 5-fluorocytosine	100
Pear (*Pyrus communis* L.)	A reporter gene was used to set-up the method	Site-specific recombination (R/*Rs*) induced by dexamethasone followed by selection on 5-fluorocytosine	100
Plum ( (Prunus domestica L.)	*	No use of marker gene	101
Apricot (*Prunus armeniaca* L.)	A reporter gene was used to set-up the method	Site-specific recombination (Cre/*LoxP*) induced by 17-β-estradiol	102
	A reporter gene was used to set-up the method	Site-specific recombination (R/*Rs*)	103
			
*GENOME EDITING*			
* Species*	*Targeted gene*	*Method*	*References*
Grapevine (*Vitis vinifera* L.)	*VvPDS* gene which confers albino phenotype	Vector containing CRISPR/Cas9+sgRNA delivered by *A.t.*	104
	*VvIdnDH* gene which controls the biosynthesis of tartaric acid	Vector containing CRISPR/Cas9+sgRNA delivered by *A.t.*	26
	VvMLO7 which confers Powdery mildew resistance	Direct delivery of purified CRISPR/Cas9 ribo-nucleoproteins to protoplast	27
Apple (*Malus* x *domestica* Borkh.)	*MdDIPM-1*, *MdDIPM- 2*, and *MdDIPM-4* which increase resistance to fire blight disease	Direct delivery of purified CRISPR/Cas9 ribo-nucleoproteins to protoplast	27
	*MdPDS* gene which confer albino phenotype	Vector containing CRISPR/Cas9+sgRNA delivered by *A.t.*	105
Orange (*Citrus sinensis* Osbeck)	Cs*PDS* gene which confer albino phenotype	Vector containing CRISPR/Cas9+sgRNA agroinfiltration	106
	Region in the promoter *CsLOB1* which decreases susceptibility to citrus canker	Vector containing CRISPR/Cas9+sgRNA delivered by *A.t.*	107
Duncan grapefruit (*Citrus paradisi* Macf.)	Region in the promoter of the gene *CsLOB1* which decreases susceptibility to citrus canker	Vector containing CRISPR/Cas9+sgRNA delivered by *A.t.*	108
	*CsLOB1* which decreases susceptibility to citrus canker	Vector containing CRISPR/Cas9+sgRNA delivered by *A.t.*	109

*The paper is reported for the relevance of the method employed for marker-gene elimination (the gene of interest used is not species-specific and transformants cannot be classified as cisgenic or intragenic).
